# Global mental health and HIV care: gaps and research priorities

**DOI:** 10.1002/jia2.25714

**Published:** 2021-06-24

**Authors:** Theresa E Senn, Gregory L Greenwood, Vasudev R Rao

**Affiliations:** ^1^ National Institute of Mental Health National Institutes of Health Rockville MD USA

**Keywords:** mental health, HIV, biological factors, implementation science, health behaviour

Common mental disorders, including depression, anxiety and post‐traumatic stress disorder (PTSD), occur at high rates among people living with HIV (PLWH) [1]. Mental disorders are associated with poorer HIV care continuum outcomes [2] and co‐occur with other psychological and structural factors including violence, stigma and other social determinants of health, which are additional barriers to HIV treatment [3]. Furthermore, mental disorders may interact with and impact biological factors to worsen HIV outcomes.

This viewpoint highlights three research gaps in mental health among PLWH: (a) understanding complex interactions between biological, psychological and structural factors that influence mental disorders in PLWH; (b) developing and testing interventions to address mental health, as well as co‐occurring psychological and structural factors, to improve HIV outcomes and (c) implementation science to understand how to best implement and scale up evidence‐based interventions to improve mental health and HIV outcomes (see Figure 1).

**Figure 1:**
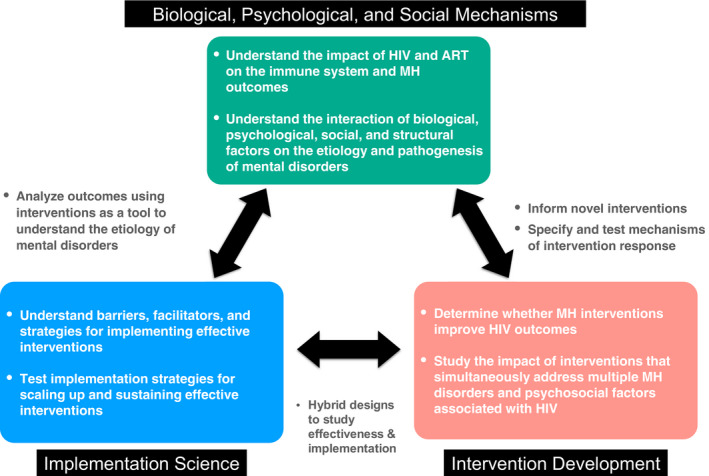
Research gaps in the study of mental health among people living with HIV. MH, mental health; PLWH, people living with HIV; ART, antiretroviral therapy.

## Understanding the complex interactions between biological, psychological/behavioural, social and structural factors that influence mental disorders in PLWH

Biological, psychological, social and structural factors interact to influence the development of mental disorders among PLWH. Both HIV and Antiretroviral therapy (ART) (e.g. efavirenz, dolutegravir) [4] cause inflammatory immune‐related changes in the Central nervous system (CNS) and the periphery, and these alterations may lead to disturbances in neurotransmitters and circuits modulating mood and behaviour [5]. The impact of HIV and ART on the peripheral immune system and neuroimmune milieu is well studied [6, 7]; however, these changes have not yet been linked to mental health outcomes seen in PLWH.

In addition, co‐occurring factors, such as stigma, access to education and living in the context of structural poverty and a fragile health system have an impact on the mental health of PLWH [8]. Few studies concurrently examine somatic mechanisms such as HIV‐ and ART‐related immune activation, neuroimmune dysfunction, microbiome alterations and metabolome variations alongside non‐somatic aetiologies such as violence, stigma and stress. Analyzing genetic, metabolomic, microbiome, neurobiological, imaging, behavioural, psychological, demographic and clinical data from long‐standing cohorts, such as the Rakai Community Cohort in Uganda [9] or South Africa’s National HIV Programme cohort [10], could lead to better understanding of the aetiologies underlying mental disorders in PLWH. In low‐resource countries where such cohort data might not be readily available, methods to capture key parameters utilizing primary care and community‐derived data should be developed.

A better understanding of complex interactions between factors at multiple levels will aid in identifying key modifiable targets to inform the development of targeted pharmacological and non‐pharmacological interventions for PLWH with mental disorders. For example research has found that adverse childhood experiences are associated with less responsiveness to some antidepressants, suggesting that experience may impact biology in a way that subsequently impacts response to depression treatment [11]. This research can be expanded to include PLWH, incorporate other psychological and social stressors and identify the mechanisms underlying differential response to treatment, to inform novel and targeted interventions.

## Developing and testing interventions to address mental health and other psychological and structural comorbidities, to improve hiv outcomes

Although studies have demonstrated the effectiveness of evidence‐based mental health interventions on mental health outcomes, the extent to which they impact HIV outcomes is less clear [12]. Studies from the United States have shown that a combined approach integrating mental health treatment with HIV care interventions (e.g. Problem Solving Therapy for depression and adherence) [13] can lead to improvements in both categories of health outcomes [14, 15], but comparable data are not widely available across low‐resource settings.

There are few empirically supported interventions that simultaneously address multiple common mental health disorders and social factors associated with HIV. One promising approach is the Common Elements Treatment Approach (CETA). This transdiagnostic intervention, consisting of common techniques underlying depression, anxiety and substance use treatment [16], improved mental health, substance use and intimate partner violence in a recent randomized controlled trial [16, 17], but needs more investigation in terms of HIV outcomes [18]. Another cross‐cutting, integrative approach in need of rigorous evaluation is the World Health Organization Mental Health Gap Action Programme (mhGAP) Intervention Guide (IG), that delivers evidence‐based screening, treatment and referral to address common mental, neurological and substance use disorders [19]. Key interventions (e.g. accelerators) that simultaneously impact multiple targets, or combine to support individual targets, could promote a holistic approach to meet wide‐ranging needs [20]. Targeting positive affect, coping and resilience is a growing area of research that could improve HIV outcomes [21]. Specifying and testing mechanisms of intervention action will be key to understanding why interventions do (or do not) work.

## Implementation science to deliver and scale up mental health interventions

Evidence‐based mental health interventions for PLWH must be implemented effectively and delivered across global contexts using scalable and cost‐efficient approaches. Researchers have begun to examine key factors impacting the delivery of mental health interventions for PLWH, including barriers and facilitators to implementation, as well as feasibility, acceptability and costs. Implementation strategies are often elicited from patients, stakeholders and experts across contexts, and tested through rigorous designs. Strong implementation science research is guided by a theoretical framework, which may inform implementation planning, barriers and facilitators to implementation, and/or implementation evaluation [22]. Hybrid designs allow for simultaneous testing of intervention and implementation outcomes.

Additional research is needed to understand the mediators through which implementation strategies impact outcomes, to improve understanding of successful (and unsuccessful) approaches and help ensure strategies are appropriately used in new settings and populations [23]. Once successful approaches have been implemented, further research is needed to ensure these approaches are sustained. Implementation strategies to address mental health in PLWH that could be studied further include: task sharing, where interventions are adapted for delivery by individuals with less advanced training [24]; mHealth approaches, to deliver interventions cost‐efficiently or in settings where transportation is a barrier [25]; differentiated care approaches, where treatment intensity, frequency or type is matched to an individual’s needs, reducing costs of providing excess or inappropriate treatment [26]; integration of mental health and HIV services [27]; and transdiagnostic interventions [16] which target a range of mental disorders. All these approaches hold promise for scaling up mental health interventions for PLWH globally. Finally, as many countries with high HIV burdens already have platforms for providing HIV treatment, these may be leveraged to share costs and more efficiently deliver mental health services to PLWH.

In conclusion, the substantial burden of mental health disorders among PLWH negatively impacts health and wellbeing. Research on the aetiology and pathogenesis of mental disorders in PLWH, intervention research that addresses both mental health and HIV care outcomes, and implementation science research to implement, scale up and sustain interventions is needed to improve physical and mental health in PLWH.

## Competing interests

The authors declare that they have no competing interests.

## Authors’ contributions

TES, GLG and VRR contributed to the conception of the manuscript, were involved in drafting the manuscript, and critically revised the manuscript. All authors have read and approved the final manuscript.
